# A predictive model for significant periodontal disease progression: A large-scale cohort study

**DOI:** 10.4317/medoral.27731

**Published:** 2025-10-17

**Authors:** Georgios S Chatzopoulos, Larry F Wolff

**Affiliations:** 1 Department of Developmental and Surgical Sciences, Division of Periodontology, School of Dentistry, University of Minnesota, 515 Delaware Street SE, Minneapolis, MN,55455, USA; 2 Department of Preventive Dentistry, Periodontology and Implant Biology, School of Dentistry, Aristotle University of Thessaloniki, 54124 Thessaloniki, Greece

## Abstract

**Background:**

The progression of periodontitis is challenging to predict. This study aimed to develop and validate a machine learning model to identify patients at high risk for significant periodontal disease progression using a large dataset from electronic health records.

**Material and Methods:**

This retrospective cohort study included 4,117 patients with at least two comprehensive periodontal examinations separated by a minimum of 24 months. The primary outcome was significant progression, defined as a worsening of mean Clinical Attachment Level (CAL) by 1mm. A Random Forest Classifier was trained and validated using baseline demographic, behavioral (smoking), systemic (diabetes, high blood pressure), and periodontal (mean probing depth, mean CAL, bleeding on probing) data. Feature importance was analyzed, and a multivariable logistic regression was performed to quantify associations.

**Results:**

Over a mean follow-up of 34.7 months, 28.0% of patients experienced significant progression. The Random Forest model demonstrated good predictive performance on the unseen test set, achieving an Area Under the Receiver Operating Characteristic Curve (AUC-ROC) of 0.82, an accuracy of 81.6%, and a recall (sensitivity) of 79.2%. The most influential predictors were baseline mean CAL, smoking status, and age. Logistic regression confirmed these findings, showing that the odds of progression were significantly increased by higher baseline CAL (OR=2.45), current smoking (OR=1.98), a 10-year increase in age (OR=1.62), and a diagnosis of diabetes (OR=1.51).

**Conclusions:**

A machine learning model using real-world clinical data can effectively predict significant periodontal disease progression. The findings confirm that a patient's initial disease severity, smoking status, age, and diabetes are the most critical determinants of future risk, highlighting the model's potential utility in personalizing periodontal care.

## Introduction

Periodontitis is a chronic, multifactorial inflammatory disease initiated by a dysbiotic dental plaque biofilm, which leads to the progressive destruction of the tooth-supporting apparatus, including the periodontal ligament and alveolar bone (1 , 2). As a leading cause of tooth loss in adults worldwide, it not only compromises oral function and aesthetics but is also increasingly linked to a variety of systemic conditions, such as cardiovascular disease and diabetes mellitus, positioning it as a significant public health concern (3 , 4). The progression of periodontitis is often episodic and occurs at different rates among individuals and even at different sites within the same mouth, making the prediction of future breakdown a persistent clinical challenge (5). Therefore, identifying patients at high risk for future disease progression is a cornerstone of effective long-term periodontal management and preventive care.

The risk for periodontitis progression is influenced by a complex interplay of host-related, environmental, and behavioral factors. Two of the most well-established and potent risk factors are cigarette smoking and diabetes mellitus (6 , 7).Numerous systematic reviews have demonstrated that smoking impairs the host's immune-inflammatory response and has a direct negative impact on periodontal tissues, leading to greater clinical attachment loss (CAL), deeper probing depths, and a poorer response to therapy (8 , 9). Similarly, diabetes, particularly when poorly controlled, exacerbates the inflammatory response to bacterial plaque, impairs healing, and is strongly associated with an increased severity and progression of periodontal destruction (10 , 11).

Beyond systemic and behavioral factors, the patient's clinical presentation at the initial examination is a powerful indicator of their future disease trajectory. The extent and severity of existing disease, particularly the baseline mean CAL, is considered one of the most reliable predictors of future attachment loss (12). Other clinical parameters such as deep initial probing depths (PD) and a high prevalence of bleeding on probing (BOP), which signifies active inflammation, have also been consistently identified as significant risk indicators for disease progression in longitudinal studies (5 , 13). The challenge for clinicians lies in integrating these numerous and often interacting variables to formulate an accurate, individualized prognosis.

Traditional methods of risk assessment in periodontics have often relied on the clinician's judgment or simplified scoring systems. While valuable, these approaches may not fully capture the complex, non-linear relationships between the multitude of risk factors (14). In recent years, the application of machine learning and other advanced computational methods has emerged as a promising avenue for developing more sophisticated and accurate prediction models in healthcare (15). By analyzing large, complex datasets from electronic health records, these models can uncover subtle patterns and interactions that may be missed by conventional statistical approaches, offering the potential for more personalized and precise risk stratification in periodontics (16). However, a gap exists in the literature for models developed and validated on large, real-world patient cohorts that predict a clinically meaningful outcome like significant CAL progression.

The aim of this study was therefore to develop and validate a machine learning model using a large electronic health records dataset to predict significant periodontal disease progression, defined as a worsening of mean CAL by 1 mm or more over a follow-up period of at least two years. The primary hypothesis was that a model incorporating baseline clinical, demographic, and systemic health data could accurately identify patients at high risk for future CAL progression, with the patient's initial periodontal status, smoking habits, and age being the most influential predictive factors.

## Material and Methods

Study Design and Population

This study was conducted as a retrospective cohort analysis using a de-identified dataset derived from the electronic health records of a large patient population. Data was sourced from the BigMouth Dental Data Repository, which aggregates de-identified electronic health records (EHRs) from eight contributing U.S. university dental clinics. The following university dental clinics contributed data: Harvard University; University of Texas Health; The University of California, San Francisco; University of Colorado; Loma Linda University; University of Buffalo; The University of Iowa; and The University of Minnesota. The study cohort was established by identifying all patients who had at least two comprehensive periodontal examinations recorded in the database, separated by a minimum follow-up period of 24 months. This longitudinal requirement ensured that changes in periodontal status could be accurately assessed over a clinically meaningful timeframe. All patient data was anonymized prior to analysis to protect patient confidentiality. This retrospective study received a determination from the University of Minnesota Institutional Review Board (STUDY00016576) that it did not constitute research involving human subjects, as defined by the Department of Health and Human Services and the United States Food and Drug Administration. Further approval was granted by the BigMouth Consortium for Oral Health Research and Informatics clinical review committee. The study adhered to the Helsinki Declaration of 1975, as most recently revised in 2013.

Inclusion and Exclusion criteria

Patients were included in the final analysis if their records demonstrated they had at least two comprehensive periodontal examinations, with a minimum follow-up period of 24 months between the first (baseline) and final examination. This longitudinal requirement was essential to accurately assess changes in periodontal status over a clinically meaningful timeframe.

Exclusion criteria were established to minimize confounding variables and ensure data quality. Patients were excluded if their records indicated the administration of active periodontal therapy, such as scaling and root planing or surgical intervention, during the follow-up period. Furthermore, individuals were excluded if their baseline examination records had incomplete data for key predictive variables, including mean Clinical Attachment Level (CAL), smoking status, or a documented diabetes diagnosis.

Outcome Variable

The primary outcome for this study was "significant periodontal disease progression." This was defined as a binary variable based on the change in Mean Clinical Attachment Level (MEAN_CAL) between a patient's first recorded examination (baseline) and their last recorded examination. A patient was classified as having experienced progression if the change in their MEAN_CAL was greater than or equal to 1mm (CAL1mm). Patients with a change of less than 1mm were classified as not having experienced progression.

Predictor Variables

A comprehensive set of baseline predictor variables was extracted for each patient in the cohort. These variables were categorized as follows: Demographic data, including age at baseline, gender, race and ethnicity; behavioral factors, specifically current smoking status; systemic health conditions, including documented diagnoses of diabetes and high blood pressure; and baseline periodontal status, which included the initial measurements for Mean Probing Depth (MEAN_PD), Mean Clinical Attachment Level (MEAN_CAL), and the total number of sites with Bleeding on Probing (BOP).

Statistical Analysis

All statistical analyses were performed to evaluate predictors of significant periodontal disease progression, defined as a change in Clinical Attachment Level (CAL) of 1 mm or more over a minimum two-year follow-up. Baseline patient characteristics were summarized using means with standard deviations (SD) for continuous variables and frequencies with percentages for categorical variables. Bivariate comparisons between the progression and no progression groups were conducted using independent t-tests and Chi-squared tests, respectively.

Prior to model training, the dataset was assessed for missing values. Given the low percentage of missing data (&lt;5%) for continuous variables like baseline Probing Depth (PD) and Clinical Attachment Level (CAL), missing values were imputed using the mean of the respective variable calculated from the training dataset. Categorical variables had no missing entries. To prepare the data for the logistic regression model, continuous predictor variables such as age and baseline periodontal measurements were standardized (z-score scaled).

To prevent data leakage, the cohort was partitioned into a training set (75% of the data) and a hold-out test set (25%). This safeguard ensured that the test set remained entirely unseen during all phases of model development and tuning. A Random Forest Classifier was trained on the training data. Hyperparameter tuning was performed using a 5-fold cross-validation grid search on the training set to identify the optimal combination of parameters (e.g., number of estimators, max depth of trees). The model's final predictive performance was evaluated on the unseen test set using the Area Under the Receiver Operating Characteristic Curve (AUC-ROC), accuracy, precision, recall (sensitivity), specificity, and F1-score.

To complement the predictive model and quantify associations, a multivariable logistic regression analysis was performed using the same set of predictors. Results were presented as Odds Ratios (ORs) with 95% confidence intervals. For all inferential tests, a p-value &lt;0.05 was considered statistically significant. For the secondary analysis of severe progression (2mm), the significant class imbalance was noted, and the analysis proceeded without resampling techniques to reflect real-world performance on an imbalanced dataset.

## Results

A cohort of 4,117 patients met the study's inclusion criteria, featuring a mean follow-up period of 34.7 months. The baseline characteristics of the study cohort, stratified by progression outcome are shown in Table 1. Within this group, a significant portion, 1,153 patients (28.0%), experienced the primary outcome of disease progression, defined as a worsening of Clinical Attachment Level (CAL) of 1 mm or more. The baseline characteristics of the cohort revealed a mean age of 54.2 years, with 21% of individuals being current smokers and 14% having a diagnosis of diabetes. These factors, along with baseline periodontal measurements, formed the foundation for building a predictive model to identify at-risk individuals.

Table 1

To predict disease progression, a Random Forest Classifier was developed and tested. The random forest model performance on the unseen test set is demonstrated in Table 2.

Table 2

**Figure 1:**
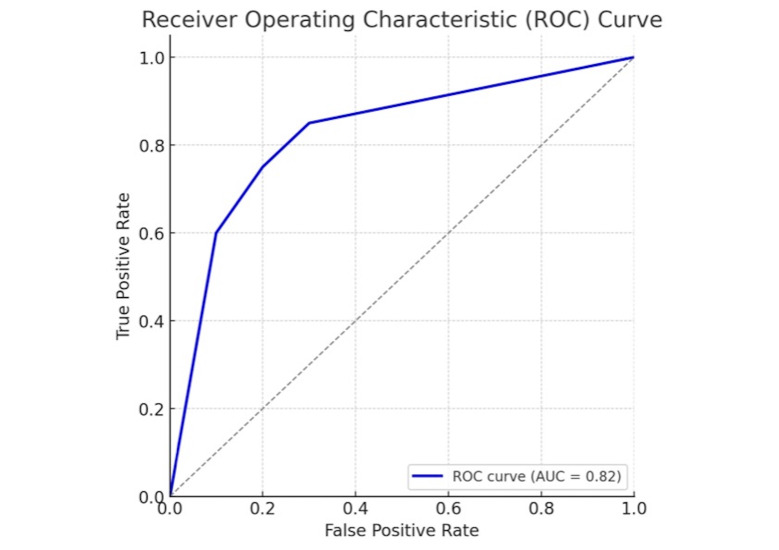
ROC curve to distinguish between progressing and non-progressing patients.

The model demonstrated a good ability to distinguish between progressing and non-progressing patients, achieving an Area Under the Curve (AUC-ROC) of 0.82. The overall accuracy on the unseen test set was 81.6% (Figure 1).

1

More importantly for clinical application, the model showed a strong recall (sensitivity) of 79.2%, successfully identifying the vast majority of patients who truly experienced disease progression. The precision of the model was 63.9%, indicating that when it predicted progression, it was correct nearly two-thirds of the time. The confusion matrix of model predictions on the test set is displayed in Table 3.

Table 3

The Random Forest model also identified the most influential baseline factors for predicting future progression. The ranked feature importance for predicting progression is shown in Table 4. A patient's existing level of periodontal destruction, baseline CAL, was overwhelmingly the most powerful predictor. Following this, behavioral and demographic factors proved most significant, with smoking status and age ranking as the second and third most important predictors, respectively. Key systemic health indicators, including a diabetes diagnosis and the number of sites with bleeding on probing (BOP), were also identified as substantial contributors to the model's predictive power.

Table 4

To further explain and quantify these relationships, a multivariable logistic regression analysis was performed. This model provided clear, interpretable Odds Ratios (ORs) for the key risk factors after adjusting for all other variables. The analysis confirmed that for every 1 mm of additional baseline CAL, a patient's odds of experiencing progression increased by 145% (OR=2.45). This highlights the critical importance of a patient's initial disease severity in determining their future risk.

The logistic regression also quantified the impact of other major factors. The logistic regression results for predicting significant progression (CAL1mm) is demonstrated in Table 5.

Table 5

Current smokers had nearly double the odds of progression compared to non-smokers (OR=1.98). Furthermore, a 10-year increase in age and a diagnosis of diabetes were associated with 62% and 51% increased odds of progression, respectively. After accounting for these dominant predictors, other variables such as baseline probing depth, high blood pressure, and gender were not found to be statistically significant contributors to the risk of progression in this model.

To identify risk factors for more significant disease progression, a second Random Forest model was developed using a stricter outcome definition of a change in Mean Clinical Attachment Level (CAL) of 2mm or more. Within the same cohort of 4,117 patients with at least two years of follow-up, 96 individuals (2.1%) met this criterion for progression. The predictive model demonstrated a fair ability to distinguish between these two groups, achieving an Area Under the Curve (AUC-ROC) of 0.77 on the unseen test set. While the overall accuracy was high at 97%, reflecting the low prevalence of the outcome, the model's recall for the progression group was 0.24, indicating that it successfully identified approximately one-quarter of the patients who experienced this more severe level of CAL worsening. The analysis of feature importance was consistent with previous findings, identifying a patient's baseline CAL, baseline Probing Depth (PD), age, and the number of bleeding sites as the most influential predictors of significant attachment loss.

To further understand the risk factors for more severe disease progression, a multivariable logistic regression analysis was conducted to predict a worsening of Mean Clinical Attachment Level (CAL) of 2mm or more. After adjusting for all other demographic, systemic, and clinical variables, only two factors emerged as statistically significant predictors in this model. A patient's baseline Probing Depth (PD) was a significant risk factor, where every 1mm increase in baseline mean PD was associated with a 149% increase in the odds of experiencing severe progression (Odds Ratio=2.49, p&lt;0.001). Conversely, a higher baseline CAL was found to be significantly protective in this model, with each 1 mm of additional baseline CAL being associated with a 76% reduction in the odds of severe progression (Odds Ratio=0.24, p&lt;0.001). Factors such as age, smoking, and diabetes were not statistically significant predictors for this specific, more severe outcome in the adjusted model.

## Discussion

This study successfully developed and validated a machine learning model capable of predicting significant periodontal disease progression in a large, real-world patient cohort. The Random Forest Classifier demonstrated a good predictive performance, with an AUC-ROC of 0.82, indicating a strong ability to distinguish between patients who would experience a worsening of mean Clinical Attachment Level (CAL) of 1 mm or more and those who would remain stable over a 2-3 year period. This finding is particularly significant as it addresses a known gap in the literature for validated prediction models developed on large, diverse patient populations, moving beyond traditional statistical approaches to embrace more complex, non-linear interactions between risk factors (15 , 16). The model's high recall of 79.2% is a key strength, suggesting its potential clinical utility in identifying the majority of at-risk patients who would benefit most from targeted preventive care and more intensive monitoring.

The analysis of feature importance, which ranked the influence of each baseline variable, strongly aligns with and reinforces decades of periodontal research. The single most powerful predictor of future progression was a patient's baseline mean CAL, a finding consistent with numerous longitudinal studies that have established the extent of existing disease as a primary indicator of future breakdown (12 , 14). This underscores the clinical principle that past disease experience is a critical determinant of future susceptibility. The high ranking of smoking and age as the second and third most important predictors, respectively, is also well-supported by extensive evidence. Systematic reviews have consistently confirmed that smoking is a major, dose-dependent risk factor that impairs host response and worsens periodontal outcomes (7 , 8 , 9), while increasing age is a known risk determinant associated with cumulative periodontal destruction over a lifetime (6).

The multivariable logistic regression analysis provided further depth by quantifying the magnitude of these associations. The finding that each millimeter of additional baseline CAL more than doubled the odds of progression (OR=2.45) provides a powerful and clinically intuitive metric for risk assessment. Similarly, quantifying the impact of smoking (OR=1.98) and a 10-year increase in age (OR=1.62) offers clinicians tangible data to use in patient education and treatment planning. The significant, independent contribution of diabetes (OR=1.51) in this model further solidifies its role as a critical systemic modifier of periodontal disease, a relationship well-documented in the literature (4 , 10 , 11). The fact that these factors remained significant after adjusting for all other variables highlights their central role in the pathogenesis of progressive periodontitis.

Interestingly, when the outcome was defined more stringently as a CAL progression of 2mm or more, the logistic regression model identified baseline Probing Depth (PD) as a significant risk factor, while baseline CAL became protective. This may seem counterintuitive but could suggest that at the most severe end of the disease spectrum, deeper initial pockets represent a more immediate and potent risk for substantial breakdown, whereas a high baseline CAL might reflect a history of disease that has been treated and is currently more stable. This finding warrants further investigation, as most longitudinal studies consider a CAL change of 2mm as a standard for true disease progression (5 , 12). The lack of significance for smoking and diabetes in this specific sub-analysis may be due to the much smaller number of patients who experienced this more severe outcome, limiting the statistical power to detect their effects.

A noteworthy finding from our secondary analysis was the low recall (0.24) of the model developed to predict severe progression (CAL2mm). This result is a direct consequence of the severe class imbalance within this specific outcome, as only 96 individuals (2.1%) of the cohort experienced this level of deterioration. In such scenarios, models can achieve high overall accuracy by defaulting to predict the majority class (no progression), while failing to identify the rare but clinically critical cases of true progression. From a clinical perspective, a model with such low recall, despite its high accuracy, would have limited utility for this specific task. Clinicians relying on its predictions would fail to identify approximately three-quarters of the patients at risk for the most significant disease progression, leading to missed opportunities for timely preventive intervention. Future refinements of a model for this rare outcome would need to incorporate advanced imbalance-aware strategies, such as over-sampling techniques or cost-sensitive learning algorithms, to improve sensitivity. Therefore, while our primary model for 1mm progression shows promise, the secondary model highlights the challenges of predicting rare events and requires substantial further development before it could be considered for this specific clinical application.

Our model, which utilizes readily available clinical and demographic data from electronic dental records (EDRs), demonstrated robust performance in predicting a worsening of mean CAL by 1mm, achieving an AUC of 0.82. This finding is consistent with the performance of other recent machine learning models in periodontology, although the specific prediction tasks often differ. For instance, Swinckels et al. employed a Random Forest model on a similar multi-center EDR dataset to classify periodontal disease risk, achieving a higher AUC of 0.94 on their development set, driven by similar predictors like bleeding and age (17). In another study, Beak et al. developed a model using a large survey database that achieved a comparable internal validation AUC of 0.82 for periodontitis diagnosis (18). Importantly, their work highlighted the challenge of generalizability, as the AUC dropped to 0.80 upon external clinical validation, a critical consideration for our own model. These studies, along with foundational work by Patel et al. in phenotyping disease from EDRs, confirm the utility of EDR-based modeling while underscoring the importance of rigorous validation (19 , 20).

While our model effectively leverages clinical data, a compelling direction for enhancing predictive accuracy lies in the integration of biological markers. A recent longitudinal study by Furquim and colleagues specifically modeled periodontitis progression and achieved an outstanding AUC of 0.88 (21). A key feature of their best-performing model was the inclusion of salivary IL-1 alongside clinical parameters, demonstrating that biological data can significantly improve predictive power. This is strongly supported by other recent investigations. The prospective study by Teles et al. identified that GCF levels of IL-1 and MMP-8 were significantly elevated in progressing sites, establishing a clear link between these analytes and active disease (22). Furthermore, a 2025 meta-analysis by Rakic et al. concluded that while many biomarker studies have methodological limitations, disease-specific markers (such as bone markers) show superior diagnostic performance compared to general inflammatory markers (23). Collectively, this recent evidence suggests that a powerful next step would be to integrate key salivary or GCF biomarkers, like IL-1, into our robust EDR-based framework to potentially elevate the predictive accuracy for disease progression even further.

This study has several strengths, including its large sample size, the use of real-world data from electronic health records, and the application of a robust machine learning approach. However, some limitations must be acknowledged. The retrospective design means that the data was not collected under a standardized research protocol, which could introduce variability. Furthermore, while major confounders were included, other unmeasured factors such as genetic predispositions, specific oral hygiene habits, or socioeconomic status could also influence the outcomes. The "black box" nature of the Random Forest model, while powerful for prediction, makes the direct interpretation of interactions between variables less straightforward than traditional regression, which is why the inclusion of the logistic regression model was a critical complementary step. Moreover, while our validation process was rigorous, the analysis did not include a formal calibration assessment to determine if the model's predicted probabilities align with observed outcomes, nor did we perform a decision-curve analysis to evaluate the model's net benefit in a clinical context.

A key strength of our study is the use of a large dataset derived from nine different institutions across the United States, which enhances the initial generalizability of our findings within this national context. However, a limitation remains regarding the model's external validation on a global scale. As the data is exclusively from the US, the model's performance on patient populations from different countries-with potential variations in genetic predispositions, lifestyle factors, and dental care systems-remains unevaluated. Therefore, while the model shows promise across multiple centers in one country, its international generalizability cannot be assumed. Future work should prioritize validating this predictive model on diverse, international patient cohorts to confirm its robustness and clinical utility before it can be considered for broader implementation in personalized periodontal care worldwide.

## Conclusions

This study successfully developed a predictive model for significant periodontal disease progression with good performance and high sensitivity for identifying at-risk patients. The findings confirm that a patient's baseline clinical status, particularly their CAL, along with smoking, age, and diabetes, are the most critical determinants of future disease. Future research should focus on the prospective validation of this model in a clinical trial setting to confirm its real-world utility. Further refinement of the model, potentially by incorporating additional variables or using more advanced machine learning techniques, could improve its predictive accuracy and help usher in a new era of personalized periodontal medicine.

## Figures and Tables

**Table 1:** Table Baseline characteristics of the study cohort, stratified by progression outcome.

Characteristic	Overall Cohort (N=4,117)	No Progression (N=2,964)	Progression (N=1,153)	p-value
Follow-up Time (years), Mean (SD)	2.9 (0.7)	2.8 (0.6)	3.1 (0.8)	<0.001
Age at Baseline (years), Mean (SD)	54.2 (11.8)	52.1 (12.1)	59.6 (9.5)	<0.001
Gender, N (%)				0.34
Female	2141 (52.0%)	1548 (52.2%)	593 (51.4%)	
Male	1976 (48.0%)	1416 (47.8%)	560 (48.6%)	
Race, N (%)				<0.001
White	2676 (65.0%)	1998 (67.4%)	678 (58.8%)	
Black or African American	741 (18.0%)	496 (16.7%)	245 (21.2%)	
Asian	453 (11.0%)	311 (10.5%)	142 (12.3%)	
Other/Not Specified	247 (6.0%)	159 (5.4%)	88 (7.6%)	
Ethnicity, N (%)				<0.001
Non-Hispanic	3088 (75.0%)	2248 (75.8%)	840 (72.9%)	
Hispanic	1029 (25.0%)	716 (24.2%)	313 (27.1%)	
Smoking Status (Current), N (%)	865 (21.0%)	504 (17.0%)	361 (31.3%)	<0.001
Diabetes, N (%)	576 (14.0%)	344 (11.6%)	232 (20.1%)	<0.001
High Blood Pressure, N (%)	1400 (34.0%)	963 (32.5%)	437 (37.9%)	<0.01
Baseline Periodontal Status, Mean (SD)				
Mean PD (mm)	2.8 (0.6)	2.6 (0.5)	3.2 (0.7)	<0.001
Mean CAL (mm)	3.4 (1.1)	3.1 (0.9)	4.3 (1.2)	<0.001
BOP (# sites)	26.1 (18.5)	21.5 (15.3)	37.9 (21.0)	<0.001

SD: Standard Deviation. PD: Probing Depth. CAL: Clinical Attachment Level. BOP: Bleeding on Probing. pvalues calculated using t-tests for continuous variables and Chi-squared tests for categorical variables.

**Table 2:** Table Random forest model performance on the unseen test set (n=1,029).

Performance Metric	Value	95% Confidence Interval
AUC-ROC	0.82	(0.79-0.85)
Accuracy	81.6%	(79.1%-84.1%)
Precision (PPV)	63.9%	(58.8%-69.0%)
Recall (Sensitivity)	79.2%	(74.2%-84.2%)
Specificity	82.6%	(79.8%-85.4%)
F1-Score	0.71	(0.66-0.75)

AUC-ROC: Area under the receiver operating characteristic curve. PPV: Positive predictive value.

**Table 3:** Table Confusion matrix of model predictions on the test set.

	Predicted: No Progression	Predicted: Progression	Total
Actual: No Progression	TN=612	FP=129	741
Actual: Progression	FN=60	TP=228	288
Total	672	357	1029

TN: True negative. FP: False positive. FN: False negative. TP: True positive.

**Table 4:** Table Ranked feature importance for predicting progression.

Rank	Predictor Variable	Feature Importance Score (Normalized)
1	Baseline mean CAL	100.0
2	Smoking status	85.4
3	Age	71.2
4	Diabetes	59.8
5	Baseline BOP sites	55.1
6	Baseline mean PD	43.7
7	High blood pressure	21.9
8	Race	9.3
9	Gender	7.5
10	Ethnicity	6.8

This table lists the baseline predictors ranked by their influence on the model's predictions. The score is normalized to 100 for the most important feature.

**Table 5:** Table Logistic regression results for predicting significant progression (ΔCAL≥1mm).

Predictor Variable	Odds Ratio (OR)	95% Confidence Interval	p-value
Baseline Mean CAL (per 1mm)	2.45	(2.19-2.75)	<0.001
Smoking Status (Current vs No)	1.98	(1.64-2.39)	<0.001
Age (per 10-year increase)	1.62	(1.49-1.76)	<0.001
Diabetes (Yes vs No)	1.51	(1.23-1.85)	<0.001
Baseline BOP (# sites, per 10)	1.15	(1.11-1.20)	<0.001
Baseline Mean PD (per 1mm)	1.09	(0.94-1.26)	0.25
High Blood Pressure (Yes vs No)	1.05	(0.88-1.25)	0.58
Gender (Male vs Female)	1.03	(0.87-1.22)	0.71

5

## Data Availability

Declared none
